# *Gentrepid* V2.0: a web server for candidate disease gene prediction

**DOI:** 10.1186/1471-2105-14-249

**Published:** 2013-08-16

**Authors:** Sara Ballouz, Jason Y Liu, Richard A George, Naresh Bains, Arthur Liu, Martin Oti, Bruno Gaeta, Diane Fatkin, Merridee A Wouters

**Affiliations:** 1 Structural and Computational Biology Department, Victor Chang Cardiac Research Institute, Darlinghurst, NSW 2010, Australia; 2 School of Computer Science and Engineering, University of New South Wales, Kensington, NSW 2052, Australia; 3 Centre for Molecular and Biomolecular Informatics, Radboud University Nijmegen Medical Centre, Nijmegen, The Netherlands; 4 School of Medical Sciences, University of New South Wales, Kensington, NSW 2052, Australia; 5 Molecular Cardiology and Biophysics Division, Victor Chang Cardiac Research Institute, Darlinghurst, NSW 2010, Australia; 6 School of Medicine, Deakin University, Geelong, VIC 3217, Australia; 7 Stanley Institute for Cognitive Genomics, Cold Spring Harbor Laboratory, 500 Sunnyside Boulevard, 11797, Woodbury, NY, USA

**Keywords:** Candidate disease gene prediction, Candidate disease genes, Mendelian diseases, Complex diseases, Genome-wide association studies, Genotype, Phenotype, Candidate gene identification, Genetic-association studies, Hypertension

## Abstract

**Background:**

Candidate disease gene prediction is a rapidly developing area of bioinformatics research with the potential to deliver great benefits to human health. As experimental studies detecting associations between genetic intervals and disease proliferate, better bioinformatic techniques that can expand and exploit the data are required.

**Description:**

*Gentrepid* is a web resource which predicts and prioritizes candidate disease genes for both Mendelian and complex diseases. The system can take input from linkage analysis of single genetic intervals or multiple marker loci from genome-wide association studies. The underlying database of the *Gentrepid* tool sources data from numerous gene and protein resources, taking advantage of the wealth of biological information available. Using known disease gene information from OMIM, the system predicts and prioritizes disease gene candidates that participate in the same protein pathways or share similar protein domains. Alternatively, using an *ab initio* approach, the system can detect enrichment of these protein annotations without prior knowledge of the phenotype.

**Conclusions:**

The system aims to integrate the wealth of protein information currently available with known and novel phenotype/genotype information to acquire knowledge of biological mechanisms underpinning disease. We have updated the system to facilitate analysis of GWAS data and the study of complex diseases. Application of the system to GWAS data on hypertension using the ICBP data is provided as an example. An interesting prediction is a ZIP transporter additional to the one found by the ICBP analysis. The webserver URL is https://www.gentrepid.org/.

## Background

The identification of genes implicated in human disease enables an understanding of disease mechanisms and is essential for the development of diagnostics and therapeutics. While genetic approaches such as linkage mapping or genome-wide association studies (GWAS) can successfully identify genomic regions linked to a particular disease, identification of the disease-causing genes is still a difficult task. For linkage analysis, genomic regions are large, as these analyses look at inheritance over several generations in a known pedigree which may have few recombination events. Genetic association studies, which do not focus on recent observable ancestry but rather look at historic recombination, have shorter disease-associated regions [1]. However, when applied at the genome-wide scale (eg. GWAS), the large number of markers used requires stringent correction for multiple testing, limiting the number of reliably identified candidate genes. Lower scoring markers may still indicate potential disease genes but may also be false positives. Analysis of this valuable but noisy data would benefit from a candidate disease gene prioritization approach [2].

A variety of publicly available systems employ various methodologies to map phenotype to genotype in order to predict or rank candidate disease genes [3,4]. Many of these systems are available as web services. Because oligogenic diseases are associated with disruption of genes that have similar functions [5], the most prevalent method employed is gene clustering [6]. Genes are clustered based on shared features, such as common domains, similar functional annotation, involvement in the same protein complex or signaling pathways, co-expression, or combinations of these [6-11].

**Figure 1:**
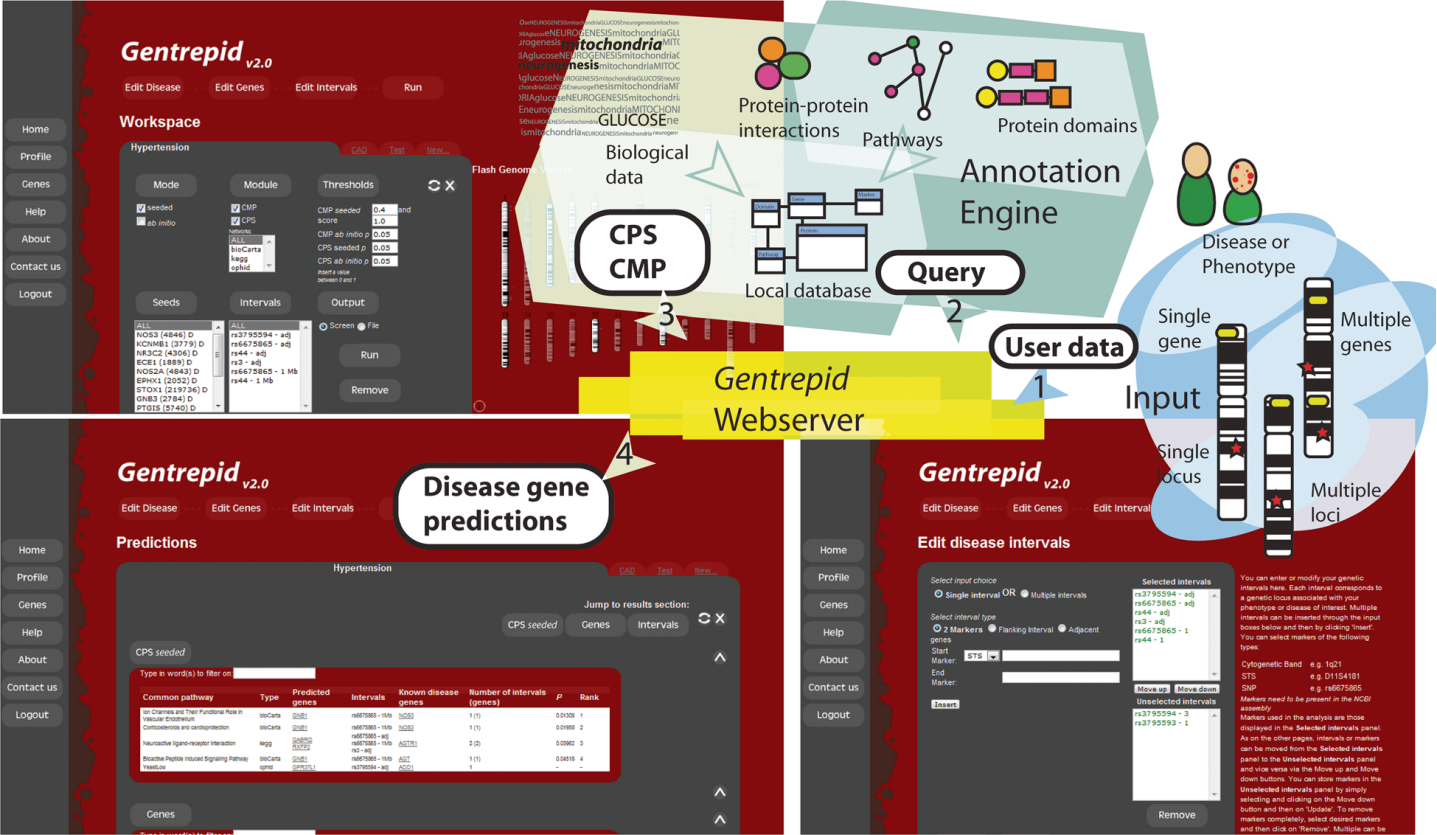
***Gentrepid*architecture and web interface overview.** Users input data as a disease locus or loci, and a phenotype, if available **(1)**. Selected loci and known genes (if applicable) are displayed on the overview page **(2)**, from which a CPS or CMP analysis can be initiated **(3)**. The interactive *Gentrepid* web server matches candidate genes against known disease genes using pre-calculated similarity scores from the annotation engine and displays the results **(4)**, or uses the *ab initio* search for enrichment of common gene annotations.

Here we present the *Gentrepid* web server, a public candidate disease gene prediction system that associates genes with specified phenotypes using genetic and biomolecular data (Figure 1). *Gentrepid* draws on two gene clustering methods to make candidate gene predictions; the Common Pathway Scanning (CPS) and Common Module Profiling (CMP) approaches [10]. Both methods identify links between genes in loci associated with a disease phenotype. CPS is based primarily on protein interaction data, whereas CMP is based on sequence data. *Gentrepid* can be assisted by phenotype-associated genes as seeds (*seeded* mode), or can work in the absence of disease gene knowledge using only phenotype-associated loci (*ab initio* mode). The system can be applied to both Mendelian and complex diseases [4,6,10].

## Construction and content

### Underlying databases and data sources

The relational database underlying *Gentrepid* was created using the PostgreSQL database management system with current biological data on human proteins and genes, including pathways, interaction data and domains. The core of the database is built on gene annotation and sequence data of the NCBI reference assembly (build 36) from RefSeq [12], with corresponding transcript (mRNA) and protein information. Protein-protein interaction (PPI) data are gathered from the Interlogous Interaction Database (I2D, formerly known as the Online Predicted Human Interaction Database, or OPHID [13]). I2D contains literature-derived data from multiple databases including BIND [14], MINT [15] and HPRD [16]. Pathway data are retrieved from BioCarta [17] and KEGG [18], which contain information on signalling and metabolic pathways respectively. Single nucleotide polymorphisms (SNPs), sequence tagged sites (STSs) and cytogenic data which are included as marker positions for input of genetic loci, are pooled from dbSNP (build 131 at the time of the analyses) [19], dbSTS and Map Viewer from NCBI [12]. Human disease gene information is extracted from the Online Mendelian Inheritance in Man (OMIM) [20] Morbid Map flat file.

### Data generation and processing

For all proteins, several sequence features are pre-calculated including signal peptide cleavage predictions by SignalP [21], coiled-coil domain predictions by Marcoil [22] and Multicoil [23], and predictions of transmembrane helices by TMHMM [24]. These data are used to provide more information about phenotype-related candidate proteins identified in the analyses. The OMIM [20] morbid map file is remediated to standardize phenotype names. In addition, simple phenotype clustering is implemented using similar text/character strings. For example, the multiple entries for Alzheimer’s disease subtypes are merged into one phenotype based on the text string “Alzheimer”. The purpose of this simple phenotype clustering is to capture as much relevant genetic information on the phenotype as possible [25]. Users have the ability to deconstruct phenotype clusters during the prediction process if desired. For the CMP algorithm, domain annotation of the proteome is performed by parsing all protein sequences against the Pfam library of Hidden Markov models [26] using HMMer [27]. Pairwise similarity scores between common domains of proteins are calculated using the Smith-Waterman algorithm implemented in SSEARCH [28].

### Quality control and updates

Data is sourced from publicly available databases that are constantly updated. In order to maintain an up-to-date version of the system, data is updated every 6 months, or when a major release of other data sources is made available. Once the data is updated, the system is benchmarked to check for consistency by running validation tests [10].

### Algorithms

#### Prior disease knowledge and the *ab initio* approach

*Gentrepid* functions under two input modes, *seeded* and *ab initio*. *Seeded* mode is assisted by phenotype-associated genes from OMIM [20] as seeds. These seed genes help define the phenotype-specific data that *Gentrepid* bases its predictions upon. This approach was shown to work successfully [10]. *Gentrepid* additionally allows candidate disease genes to be prioritized across multiple disease-associated loci in the absence of known disease genes (*ab initio* mode). In this mode, common domains and common pathways linking candidate disease genes from different loci are determined, and the degree of overrepresentation of specific domain combinations or pathways across disease-associated loci is used to make predictions. Candidate genes are then ranked according to the probability that these overrepresented domain combinations or pathways would occur randomly. The *ab initio* approach is especially useful when little is known about the disease phenotype, and can potentially discover novel biological mechanisms underpinning the disease.

#### Common pathway scanning

The Common Pathway Scanning (CPS) approach is a Systems Biology method that is based on the finding that genes for a specific phenotype are more likely to interact with other phenotype-specific genes. More specifically, these phenotype-specific proteins are involved in the same pathway or complex [5,9,29]. Utilising this knowledge, potential disease genes are predicted by searching implicated disease loci for proteins that are part of the same pathway or complex.

The CPS algorithm in *seeded* mode uses the phenotype-specific disease genes to associate pathways with the phenotype [10]. In *seeded* mode, genes within the candidate locus are checked for their occurrence in pathways associated with the seed gene. For each disease, pathways are ranked by the significance of the association, as determined by the Fisher’s exact test statistic. The *p*-value is calculated by partitioning genes in the phenotype-associated loci based on whether they are associated with the pathway in question or not [4]. Candidate genes are filtered and prioritized based on the lowest *p*-value score of the pathway they share. The default significance threshold is *p*_*path*_ < 0.05.

The CPS algorithm in *ab initio* mode requires multiple associated loci as input. In this mode, the candidate loci are searched for genes participating in common pathways. These common pathways are then ranked according to the disease-relevance of their associated pathways in the same manner as in *seeded* mode.

#### Common module profiling

The second approach, Common Module Profiling (CMP), distinguishes *Gentrepid* from other candidate disease gene prediction approaches, as it is based on the use of sequence-based domain similarity. Predicated on the principle that similar diseases are often caused by families of genes with related functions [30], it uses domain-based comparative sequence analysis to identify proteins with potential functional similarity. A domain-based comparison of proteins has several advantages over comparison of the full-length amino acid sequence of proteins. Because structure is conserved over sequence, domain-based sequence comparison searches have been shown to be more accurate than full-length sequence searches [31], improving the sensitivity of the candidate gene search. Dissection of a protein into domains provides a more fine-grained approach to identifying aspects of protein function affected in the disease. Using the Pfam library of Hidden Markov models [26], domains can be assigned to approximately 69% of human proteins which allows functional inference for around 54% of human genes. While some other web tools reference protein domains [7,11], only *Gentrepid* uses domain sequence comparison rather than keyword matching. As a result, relationships between similar but differently labelled domains can also be captured.

The CMP algorithm in *seeded* mode uses pairwise alignments of Pfam domains common to the candidate gene and the known disease gene. Candidate genes are scored based on their domain similarity to a known disease gene, and prioritized according to this score [10].

In *ab initio* mode, the CMP algorithm predicts and prioritises genes in a different manner. In this method, domain combinations are tested for over-representation in the associated loci compared to the genome as a whole through upper and lower significance tests [10]. Results are prioritized based on χ^2^_*min*_ which has been shown to give a better estimate of the significance [4].

### Webserver

#### Data input

Data input is a relatively straightforward step-wise process. All that is required is at least one phenotype-associated locus and a known disease gene, or multiple phenotype-associated loci. *Gentrepid* allows disease intervals to be specified by STSs, SNPs or cytogenetic bands in one of two ways: two markers specifying a start and stop site can be entered; or a central marker and an interval width can be used (Figure 1, bottom right panel). Both new diseases and new disease genes not currently included in the underlying *Gentrepid* database can be added by the user and incorporated in the analysis. New phenotypes may be created by the user if desired, in which case existing OMIM data will not be accessed. New disease genes are entered into the system by selecting a HUGO gene name or Entrez ID. Additionally, any of *Gentrepid*’s pre-loaded phenotype-associated genes can be deselected for the current project. As some phenotypes described in *Gentrepid* reference more than one OMIM ID, genes that an individual researcher believes to be anomalous can be removed from the project. Usage of *Gentrepid* is outlined in a help page reachable from the start page.

#### GWA input upgrade

The system was originally designed for input from linkage-mapping loci determined for Mendelian diseases. Such investigations generally involve relatively few loci, making it feasible to manually enter each locus separately into the web server. This applies similarly to the investigation of relatively small numbers of loci associated with highly significant SNPs in GWAS of complex diseases. However, candidate disease gene prediction tools such as *Gentrepid* are particularly useful when applied to larger numbers of loci. For example, when lower significance thresholds are applied to the GWAS SNPs in order to discover candidates from weaker associations [4]. To facilitate this, in version 2.0 of the system, users can enter multiple loci simultaneously by pasting a list into the text box.

#### Data retrieval

Once a phenotype and an interval, or multiple intervals, have been entered, *Gentrepid* displays the known disease genes and the genes in the selected regions, both graphically and in tabular format (Figure 1, top left panel). The graphical display utilizes Flash GViewer, a freely available flash program developed by the GMOD project (http://gmod.org/wiki/Flash_GViewer). It shows an overview of the genome, highlighting the individual genes and intervals on the chromosomes that have been selected for the project. From this page, users can choose to perform a CPS or CMP candidate disease gene analysis.

For the CPS analysis, the user can select which preloaded genes and intervals from OMIM to include in the analysis, as well as which pathway databases to include. By default all genes, intervals and pathway databases are used. The analysis is performed, and a list of candidate disease gene predictions is displayed in table format, along with the interval, the known disease genes located in the same pathway upon which the prediction was made, and the pathway source database (Figure 1, bottom left panel).

For the CMP analysis, users have the option of selecting genes and intervals to include as for CPS, and may choose the cut-off scores for the domain comparisons. The results page displays a table of predicted candidate disease genes and their locations, along with the disease gene(s) with similar domains.

For both CPS and CMP, further information about genes of interest can be found on the gene information pages, which can be accessed by clicking on the hyperlinked gene names on the results page. Gene pages are compilations of in-house bioinformatic analyses of gene products, with protein isoforms displayed under individual tabs. Protein domains, coils, and transmembrane helices are all displayed as an image, with links to the text-based results of the Pfam [27], SignalP [21], Marcoil [22], Multicoil [23], and TMHMM [24] output. The mRNA sequence can also be accessed, and if applicable, current protein isoform sequences. Both are in FASTA format. The gene page links to relevant entries in the Entrez Gene [12] and GeneCards [32] webservers, as well as the UCSC Genome Browser [33].

#### User account-based project management

*Gentrepid* can be accessed in two ways: immediately through Quicksearch or via login after registering for an account. Both are accessible via the homepage. With Quicksearches, results are generated and immediately displayed. Quicksearch results can be saved locally by the user, but they are not stored on the webserver. In order to save results on the webserver for future use, a login account is required. Login accounts are available free of charge to users from academic institutions or non-profit research institutes. They allow users to save their input and access their project at a later date or from a different client, enabling user mobility and collaboration. With an account, users can also create, edit or delete multiple projects. User data are kept in individual password-protected PostgreSQL databases and are not accessible by other accounts. Data transfer to and from the website is also encrypted and protected by a security certificate. Projects remain available until explicitly removed by the user, and can be accessed through tabs along the top panel. Data input and retrieval are analogous to the Quicksearch version.

## Utility and discussion

The system can be used on both linkage and genome-wide association data. Linkage data from family studies associates much larger regions of the genome with the disease or phenotype. Similar but not identical to an enrichment analysis, *Gentrepid*’s modules can be employed to sift through these regions and select genes that warrant further study. In the case of GWAS data, the tool can be used to search the genetic data holistically for affected pathways or molecular mechanisms. GWAS use SNPs in linkage disequilibrium (LD) to pinpoint phenotype-associated areas of the genome. When employing *Gentrepid*, genes within the vicinity of the associated SNP, not necessarily in LD, are analysed. The associated SNPs may be identifying haplotype blocks which contain control regions affecting the distal protein coding gene in *cis*. Other systems such as GRAIL [34] allow users a similar flexibility by requesting either an interval, a gene list or a set of SNPs.

The usefulness of each module for candidate gene prediction seems to depend on the heritability of the phenotype. For Mendelian diseases, CPS is the more effective of the two modules. Because of the small number of loci typically involved in Mendelian disease, it works best when there is some prior knowledge of the genes underlying the phenotype. For complex diseases, where it is believed multiple genes are involved, both modules, common pathways and common functional domains, appear to be effective. Provided enough significant loci are supplied, significant predictions can be made for complex diseases either with or without prior knowledge [4].

### Previous validation

Validation studies of the *Gentrepid* approach have been published previously for both Mendelian diseases [10] as well as complex diseases [6]. To summarize, for Mendelian diseases benchmark tests on a set of 170 disease genes for 29 diseases showed that the CMP and CPS methods have a combined sensitivity of 0.52 and a specificity of 0.97, and reduce the candidate list by 13-fold [10]. These tests were performed using artificially constructed loci of 50, 100 or 150 genes around the target disease genes, numbers which are typical of linkage-based disease loci.

Complex diseases are more difficult to benchmark due to limited knowledge of the underlying genetics. In a comparison using loci previously determined by linkage analysis against 11 highly significant Type 2 Diabetes GWAS SNPs, *Gentrepid* had a sensitivity of 0.18 and a specificity of 0.96 while reducing the candidate list by 19-fold [6]. In a second benchmark on seven complex diseases using GWAS SNP data, *Gentrepid* was capable of extracting known disease genes and predicting plausible novel disease genes in known and novel loci. Depending on the size of the search space used, the system had a sensitivity that ranged between 0.09 and 1, specificity between 0.55 and 1, and enrichment ratios up to 25-fold [4].

In addition to these benchmark tests, we have used *Gentrepid* in-house to successfully predict a novel gene for autosomal recessive spondylocostal dysostosis [35] and analyse mutations in *MESP2*, *LNFG* and *HES7*[36]. An advantage of *Gentrepid*’s biological clustering approach is that, as it does not use machine learning algorithms, it does not rely on training data sets and thus avoids concomitant problems such as model overfitting [37].

### Case study: hypertension and blood pressure

Hypertension (HTN) is a medical condition where blood pressure in the arteries is significantly elevated resulting in increased risk for cardiovascular disease, kidney disease or stroke. To demonstrate the application of the *Gentrepid* system in the analysis of GWAS data, we ran our analyses on a set of 29 significantly associated loci from a meta-GWAS performed by the International Consortium for Blood Pressure (ICBP) [38-40] where the phenotypes investigated were hypertension (HTN), systolic blood pressure (SBP), and diastolic blood pressure (DBP). The study reported 45 likely candidate and confirmed disease genes (Additional file 1: Table S1). We collated a set of 23 HTN-implicated genes from OMIM as seeds (Additional file 1: Table S2), using only genes known prior to the publication of GWAS so as not to skew our results. The seed genes are involved in pathways that regulate blood pressure and blood volume such as: calcium signalling, the renin-angiotensin system, and hormone metabolism [41-43]. Using the 29 significant loci reported, we generated a gene search space based on SNP-gene proximity labelled the *adjacent* approach where we pool genes adjacent to the associated SNP both upstream and downstream on the + and - strands [4]. We also generated a second gene search space based on SNP-gene distance, labelled the *bystander* approach. We used a 1Mbp window centred on each SNP and pooled genes within that interval. We then ran *Gentrepid* in *seeded* mode, with the 23 seed genes; and in *ab initio* mode, where no additional genotype/phenotype information is used. We report the predictions made by *Gentrepid*. We compared these predictions to the reported candidates from the study; which themselves, may or may not be the causal gene. Predictions different to the reported genes in the ICBP study [38] are annotated as alternate predictions.

From the 29 loci implicated by the ICBP study [38], the *adjacent* mapping generated a search space of 77 genes, as some loci mapped to fewer than 4 genes. From these 77 genes, *Gentrepid* returned 19 gene predictions for 15 of the 29 loci (Table 1). Of these 19 predictions, 12 were gene candidates reported by the ICBP study to be the likely disease genes. *Gentrepid* thus made 7 alternate gene predictions in 7 loci. Of these alternate predictions, many are in generic signalling pathways (eg. MAPK signalling, *p* = 0.08). Although the mitogen-activated protein kinase (MAPK) cascade is involved in various cellular functions including vascular oxidative stress, it is debated whether this is solely a symptom of hypertension [44]. The study suggests that variant alleles of the MAPK signalling pathway predispose individuals to hypertension.

**Table 1:** **Comparison of reported genes and candidate disease genes predicted by *Gentrepid *using the gene search space from the *adjacent *mapping**

**Reported SNP**	**Reported gene(s) by study**	**Predicted candidate gene(s)**	**Method**	**Common property used in prediction**	**Seed gene**	**Score**	**Relative rank**
rs419076	*MDS1*, *EVI1*	*EVI1*	CMP-ab	zf-C2H2	-	S = 5.17	3
rs13107325	*SLC39A8*	*SLC39A8*	CMP-ab	Zip	*-*	S = 150.96*	1
		*NFKB1*	CPS-s	Corticosteroids & cardioprotection	*NOS3*	P = 0.09	1
rs13139571	*GUCY1A3*,	*GUCY1A3*,	CPS-ab	Purine metabolism	*-*	P = 0.23	7
	*GUCY1B3*	*GUCY1B3*					
			CPS-s	Long-term depression	*NOS2A,*	P = 0.39	7
					*NOS3*		
rs4373814	*CACNB2*	*SLC39A12*	CMP-ab	Zip	*-*	S = 150.96*	1
rs932764	*PLCE1*	*PLCE1*	CPS-ab	Calcium signaling pathway	*-*	P = 0.31	11
			CPS-s	Calcium signaling pathway	*AGTR1,*	P = 0.31	5
					*NOS2A,*		
					*NOS3*		
rs7129220	*ADM*	*AMPD3*	CPS-ab	Purine metabolism	*-*	P = 0.23	9
rs2521501	*FURIN, FES*	*FES*	CMP-ab	Pkinase_Tyr	*-*	S = 13.97	2
rs17608766	*GOSR2*	*WNT9B*	CPS-ab	Basal cell carcinoma	*-*	P = 0.05*	3
rs6015450	*GNAS, EDN3, ZNF831, MRPS16P*	*C20orf174*	CMP-ab	zf-C2H2	*-*	S = 5.17	3
rs3774372	*ULK4*	*CTNNB1*	CPS-ab	Cell to Cell Adhesion Signaling	*-*	P = 0.01*	1
rs1458038	*FGF5*	*FGF5*	CPS-ab	MAPK signaling pathway	*-*	P = 0.08	5
		*PRDM8*	CMP-ab	zf-C2H2	*-*	S = 5.17	3
rs1813353	*CACNB2*	*CACNB2*	CPS-ab	MAPK signaling pathway	*-*	P = 0.08	5
rs17249754	*ATP2B1*	*ATP2B1*	CPS-ab	Calcium signaling pathway	*-*	P = 0.31	11
			CPS-s	Calcium signaling pathway	*AGTR1,*	P = 0.31	5
					*NOS2A,*		
					*NOS3*		
rs1378942	*CYP1A2, CSK*	*CYP1A2*	CMP-s	p450	*CYP3A5*	S = 0.15	1
		*CSK*	CPS-s	PPIN	*ACSM3,*	-	-
					*ADD1*		
rs12940887	*ZNF652*	*ZNF652*	CMP-ab	zf-C2H2	*-*	S = 5.17	3

All predictions are reported. CMP/CPS s: *seeded*. ab :*ab initio*. Score (S) or p-value (P) reported for genes predicted. Significant scores or p-values are marked with an *. A dash (−) indicates no prediction. Relative rank is the rank of the gene in the method used. For PPIN, no rank has been assigned.

The *1Mbp bystander* approach generated a search space of 386 genes. *Gentrepid* returned predictions for 25 of the 29 implicated loci, with a total of 108 gene predictions (results not shown). Of the 25 loci with predictions, 15 gene predictions were congruent with those reported by the ICBP study. Alternate predictions of note include the nuclear factor NF-kappa-B (*NFKB1*), predicted via the “Corticosteroids and cardioprotection” pathway (*p* = 9.99e-5). Corticosteroids exert a variety of actions by binding to the glucocorticoid receptor and may play a role in increased water excretion in the kidneys to reduce blood volume and atrial pressure [45]. In addition to the ZIP domain ion transporter *SLC39A8* reported by the ICBP, *Gentrepid* CMP predicted the solute carrier *SLC39A12* that, like *SLC39A8*, has a Zinc transporter domain (Zip, Pfam PF02535). The ICBP study postulated that *SLC39A8* may play a role in disease through its transport of cadmium [46,47].

As demonstrated, both SNP/gene mapping approaches are useful to obtain likely candidates. We recommend the *adjacent* approach as a first step, and then the 1Mbp *bystander* approach can be used to generate possible candidates further from the associated locus.

### Limitations and future directions

*Gentrepid*, as with all tools in the field, is limited by the current knowledge database. In an effort to compensate for the missing data, *Gentrepid* uses multiple modules (CPS, CMP) which gather data from multiple sources (protein-protein interactions, pathways, protein domains). Other protein function data that has been employed include tissue-specific expression levels [48-50], post-translational modifications [51], phylogenetic lineage [52], and other ontological classifications [53]. With the publication of regulatory annotation data such as those from the ENCODE project [54], a future direction for the tool would be to use such regulatory information as a likely candidate disease gene prediction module. Genes targeted by common transcription factors, or miRNAs, amongst other elements that affect gene expression, have also been disease associated and would be a useful update to the system [55,56].

## Conclusions

The *Gentrepid* web server facilitates the prediction and prioritization of candidate disease genes for both Mendelian and complex diseases using two complementary approaches, namely Common Pathway Scanning and Common Module Profiling. The ability to apply different approaches separately enables the application of different prioritization strategies to different categories of disease—for instance, the optimal approach for the prioritization of candidate disease genes for Mendelian diseases might differ from that for complex diseases, or for cancer. The alternate predictions made by *Gentrepid* for hypertension and blood pressure traits are interesting candidates that require further validation. The system has shown to be capable of both replicating known or reported candidates and also making novel plausible predictions, demonstrating the usefulness of *Gentrepid* in *de novo* analysis and reanalysis of GWAS data. In the future, the addition and integration of yet more data types will further increase the utility of *Gentrepid* in candidate disease gene prediction and prioritization, for all types of diseases.

## Availability and requirements

*Gentrepid* is available at https://www.gentrepid.org. It requires no special or additional data sources, other than the input data (genetic loci) from the user.

## Abbreviations

BP: Blood pressure; CPS: Common Pathway Scanning; CMP: Common Module Profiling; DBP: Diastolic blood pressure; GWAS: Genome-wide association studies; HTN: Hypertension; LD: Linkage disequilibrium; PPI: Protein-protein interactions; OMIM: Online Mendelian Inheritance in Man; SBP: Systolic blood pressure; SNP: Single nucleotide polymorphism; STS: Sequence tag sites.

## Competing interest

The authors declare that they have no competing interest.

## Authors’ contributions

SB updated and validated V2.0. JYL developed the database, made the webpages and wrote the database queries. NB supervised construction of the database. AL set-up the webserver. RAG and MAW supervised construction of the website. SB, MO and MAW wrote the manuscript. MO worked on the CMP predictions. BG, DF and MB participated in the design of the study. MAW conceived the study, participated in its design and reviewed the results from the data analysis. All authors read and approved the final manuscript.

## Supplementary Material

Additional file 1: Table S1. Novel implicated loci, and reported candidate genes from the ICBP study. **Table S2.** OMIM hypertension associated genes used as seeds for the *seeded* disease gene approach. Click here for file
