# Supplementary material for: Gentrepid V2.0: a web server for candidate disease gene prediction
Source: BMC Bioinformatics. 2013 Aug 16;14:249. doi: 10.1186/1471-2105-14-249 (PMC3844418; doi:10.1186/1471-2105-14-249)
Supplement: Additional file 1: Table S1. — Novel implicated loci, and reported candidate genes from the ICBP study. Table S2. OMIM hypertension associated genes used as seeds for the seeded disease gene approach. [file 1471-2105-14-249-S1.docx]

Supplementary Table 1 - Novel implicated loci, and reported candidate genes from the ICBP study

| **Reported  SNP** | **Chr** | **Gene locus** | **Candidate gene** | **Systolic blood pressure (SBP) SNP *P*-value** | **Diastolic blood pressure (DBP) SNP *P*-value** | **Hypertension (HTN)  SNP *P*-value** |
| --- | --- | --- | --- | --- | --- | --- |
| ***Novel*** |  |  |  |  |  |  |
| rs2932538 | 1 | *MOV10* | *MOV10*, *CAPZA1*, *ROCK* | 1.2E-09 | 9.90E-10 | 2.90E-07 |
| rs13082711 | 3 | *SLC4A7* | *SLC4A7* | 1.50E-06 | 3.80E-09 | 3.60E-04 |
| rs419076 | 3 | *MECOM* | *MDS1, EVI1* | 1.80E-13 | 2.10E-12 | 3.10E-04 |
| rs13107325 | 4 | *SLC39A8* | *SLC39A8* | 3.00E-14 | 2.30E-17 | 4.90E-07 |
| rs13139571 | 4 | *GUCY1A3-GUCY1B3* | *GUCY1A3, GUCY1B3* | 1.20E-06 | 2.00E-10 | 2.50E-05 |
| rs1173771 | 5 | *NPR3-C5orf23* | *NPR3* | 1.80E-16 | 9.10E-12 | 3.20E-10 |
| rs11953630 | 5 | *EBF1* | *EBF1* | 3.00E-11 | 3.80E-13 | 1.70E-07 |
| rs1799945 | 6 | *HFE* | *HFE* | 7.00E-12 | 1.50E-15 | 1.80E-10 |
| rs805303 | 6 | *BAT2-BAT5* | *BAT3* | 1.50E-11 | 3.00E-11 | 1.00E-10 |
| rs4373814 | 10 | *CACNB2(5′)* | *CACNB2* | 4.80E-11 | 4.00E-10 | 8.50E-08 |
| rs932764 | 10 | *PLCE1* | *PLCE1* | 7.10E-16 | 8.10E-07 | 9.40E-09 |
| rs7129220 | 11 | *ADM* | *ADM* | 3.00E-12 | 6.40E-08 | 1.00E-03 |
| rs633185 | 11 | *FLJ32810-TMEM133* | *FLJ32810, PGR, TMEM133* | 1.20E-17 | 2.00E-15 | 5.40E-11 |
| rs2521501 | 15 | *FURIN-FES* | *FURIN, FES* | 5.20E-19 | 1.90E-15 | 7.00E-07 |
| rs17608766 | 17 | *GOSR2* | *GOSR2* | 1.00E-10 | 1.70E-02 | 8.00E-02 |
| rs1327235 | 20 | *JAG1* | *JAG1* | 1.90E-08 | 1.40E-15 | 4.60E-04 |
| rs6015450 | 20 | *GNAS-EDN3* | *GNAS, EDN3, ZNF831, MRPS16P* | 3.90E-23 | 5.60E-23 | 4.20E-14 |
| ***Previously implicated*** | | | | | | |
| rs17367504 | 1 | *MTHFR-NPPB* | *MTHFR, CLCN6 NPPA/B* | 8.70E-22 | 3.50E-19 | 2.30E-10 |
| rs3774372 | 3 | *ULK4* | *ULK4* | 3.90E-01 | 9.00E-14 | 1.80E-01 |
| rs1458038 | 4 | *FGF5* | *FGF5* | 1.50E-23 | 8.50E-25 | 1.90E-07 |
| rs11191548 | 10 | *CYP17A1-NT5C2* | *CYP17A1, AS3MT* | 2.60E-12 | 2.30E-15 | 6.20E-10 |
| rs1813353 | 10 | *CACNB2(3′)* | *CACNB2* | 4.00E-12 | 1.30E-12 | 9.80E-09 |
| rs4590817 | 10 | *C10orf107* | *C10orf107* | 6.90E-26 | 9.40E-13 | 1.40E-05 |
| rs381815 | 11 | *PLEKHA7* | *PLEKHA7* | 5.30E-11 | 5.30E-10 | 3.40E-06 |
| rs10850411 | 12 | *TBX5-TBX3* | *TBX3, TBX5* | 1.80E-18 | 1.20E-14 | 1.00E-14 |
| rs17249754 | 12 | *ATP2B1* | *ATP2B1* | 3.80E-18 | 3.60E-25 | 2.60E-06 |
| rs3184504 | 12 | *SH2B3* | *SH2B3* | 5.40E-08 | 5.40E-10 | 5.20E-06 |
| rs1378942 | 15 | *CYP1A1-ULK3* | *CYP1A2, CSK* | 5.70E-23 | 2.70E-26 | 1.00E-08 |
| rs12940887 | 17 | *ZNF652* | *ZNF652* | 1.80E-10 | 2.30E-14 | 1.20E-07 |

For full table, refer to [[35](#_ENREF_35)]

Supplementary Table 2 - OMIM hypertension associated genes used as seeds for the *seeded* disease gene approach

| **Genes (HUGO)** | **Gene Entrez IDs** | **OMIM IDs** |
| --- | --- | --- |
| *HSD11B2, NR3C2, PNMT, AGTR1, PTGIS, NPR3, BMPR2, ACSM3, KCNMB1, ADD1, AGT, ECE1, GNB3, RETN, NOS3, NOS2A, CYP3A5, CYP11B2, CPS1, SELE, ATP1B1, RGS5, and EPHX1* | 3291, 4306, 5409, 185, 5740, 4883, 659, 6296, 3779, 118, 183, 1889, 2784, 56729, 4846, 4843, 1577, 1585, 1373, 6401, 481, 8490, 2052 | 145500, 108962, 124080, 125853, 145505, 178600, 189800, 218030, 265380, 605115, 608622 |
